# Modeling the Building Blocks of Biodiversity

**DOI:** 10.1371/journal.pone.0056277

**Published:** 2013-02-27

**Authors:** Lucas N. Joppa, Rich Williams

**Affiliations:** 1 Computational Ecology and Environmental Sciences, Microsoft Research, Cambridge, United Kingdom; 2 Quid.com, San Francisco, California, United States of America; Centre National de la Recherche Scientifique, France

## Abstract

**Background:**

Networks of single interaction types, such as plant-pollinator mutualisms, are biodiversity’s “building blocks”. Yet, the structure of mutualistic and antagonistic networks differs, leaving no unified modeling framework across biodiversity’s component pieces.

**Methods/Principal Findings:**

We use a one-dimensional “niche model” to predict antagonistic and mutualistic species interactions, finding that accuracy decreases with the size of the network. We show that properties of the modeled network structure closely approximate empirical properties even where individual interactions are poorly predicted. Further, some aspects of the structure of the niche space were consistently different between network classes.

**Conclusions/Significance:**

These novel results reveal fundamental differences between the ability to predict ecologically important features of the overall structure of a network and the ability to predict pair-wise species interactions.

## Introduction

Ecological networks describe who interacts with whom in ecological communities [1]. Subsets of these networks where a single type of interaction is considered, such as mutualistic interactions between plants and their pollinators [2], or antagonistic interactions between parasites and their hosts [3], are considered to be the true ‘building blocks’ of biodiversity [2]–[5]. These ‘building blocks’ provide vital ecosystem services [6], and understanding the way they are structured is important for gaining insight into everything from ecosystem function [7]–[10], to the response of ecosystems to species extinctions [11], [12], invasions [13], [14], and the influence of keystone species [15].

Theory suggests that the architecture of mutualistic and antagonistic species interaction networks should differ [2], [3]. Empirical evidence supports this, and highlights structural differences between these two types of networks in ways important to their stability and fragility [2], [3]. Moreover, there is a practice of using different structural models for mutualistic [16]–[19] and multi-trophic networks (i.e., ‘food webs’) [20]–[22], even though doing so is a purely methodological convenience unrelated to the true nature of species interactions [23]. A simple structural model capable of replicating these different types of networks would provide a single conceptual framework for linking these ‘building blocks’ in studies of population dynamics [24], ecosystem regime shifts [25], and other basic and applied ecological questions.

Here we fit a simple, probabilistic niche-structured model [26], [27] to 151 empirical networks to replicate the link structure of three different classes of bipartite ecological networks (mutualistic: n = 67, parasitic – antagonistic: n = 40, herbivory – antagonistic: n = 44). Further details on the datasets analyzed have been published previously [27]–[29]. While the structure of these classes of networks differs [2], [30], the rational for a general model is simple: since a simple one-dimensional niche axis can consistently explain the structure of multi-trophic level food webs [20], [31], one might also expect it to explain the structure of antagonistic plant-animal networks derived from these networks. Moreover, just as body size is a common explanatory trait for niche position in “food web” antagonistic networks [32], interactions in networks of mutualistic relationships like plants and their pollinators are often described as being controlled by one or a few traits in both the plant and the pollinator (e.g. length of flower corolla pollinator proboscis [33]). These examples suggest that a low-dimensional niche-structured model might explain much of the structure of all three classes of networks. The objective of this paper is to address whether or not such a low-dimensional model sufficiently explains the structure of ecological networks.

## Model

### Bipartite Probabilistic Niche Model

We developed a simple extension of the probabilistic niche model [26], [27] (PNM) for bipartite networks. Our model is complementary to one proposed earlier [34] by making use of a niche axis on which each species is positioned and where each species interacts preferentially with species nearby on this axis. That ours is a probabilistic version brings it in line with another recent model [35], although our model differs in two major ways as our unit of interaction is the species while [35] considers individuals, and our interaction function is much more general.

**Figure 1:**
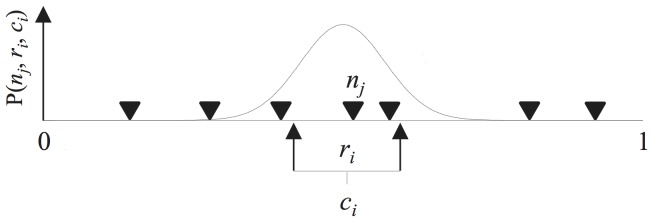
Diagram of the probabilistic niche model for bipartite networks. Each resource species has a single parameter *n_j_*; each consumer species has two parameters, *c_i_* giving the location on the niche axis where it has the highest probability of interacting with a resource species and a distribution width *r_i_*. The probability that consumer species *i* interacts with resource species *j* is defined by the probability P(*n_j_*, *r_i_*, *c_i_*), here a Gaussian.

By freeing up the constraints on niche parameters imposed by the original niche model [20], the PNM provides significantly better fit to empirical food web data than previous models, and allows for analysis of the resulting niche-related model parameters. In our bipartite probabilistic niche model (BPNM; Figure 1) each resource is placed on a niche axis and the probability that a consumer interacts with a resource is a function of the relative distance between the resource’s position on the axis and the position of the consumer’s niche on that axis. We consider bipartite networks with L links between 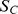 consumer species and 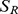 resource species. The bipartite probabilistic niche model defines the probability of interaction between resource species 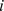 and consumer species 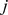. The resource species are placed on a one-dimensional niche axis; the niche position of resource species 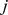 is defined as 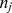. Each consumer species 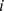 has two traits, its niche center 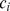 and its niche range 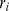. Figure 1 shows a one-dimensional version of this model. The probability of consumer 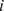 interacting with resource 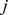 is a function of the relative distance between the resource’s niche position and the consumer’s niche center:
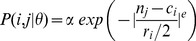
(1)where 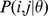 is the probability that consumer species 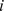 interacts with resource species 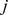 given a particular parameter set 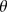 where 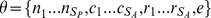; the parameter 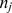 is the niche position of resource species 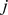; the parameter 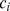 is the center of the niche of consumer species 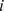; the parameter 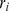 is the width of the niche of consumer species 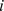; the parameter 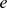 varies the cutoff rate of the niche probability function (for larger values, the niche probability function is flatter in the center and cuts off more quickly at the edges of the niche); and the parameter 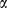 is the probability that 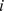 eats 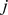 when 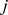 is exactly on 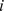
’s niche optimum (i.e. when 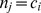).

#### Statistical methods

The set of model parameter values for a network with 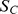 consumer species and 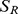 resource species is given by 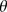, while 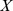 is the data, i.e., 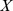 is an 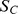
×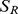 connection matrix containing an observation 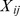 for each link 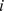, 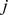 (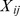 = 1 means 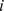 interacts with 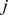; 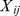 = 0 means 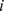 does not interact with 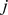). The log-likelihood is defined as:
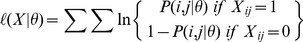
(2)


In each of the models we use, every possible interaction between species pairs is assigned a probability of occurring. This allows model performance to be calculated in a straightforward likelihood framework, and for all models, we find the maximum likelihood parameter values for each empirical network. Using likelihoods to evaluate model performance effectively separates the model’s performance evaluation from any summary metrics of network structure, a distinction that we will show to be extremely important.

### Potential Overparameterization

For all models we use simulated annealing [36] to find the maximum likelihood parameter set given the observed feeding relationships. The number of parameters in the BPNM scales as 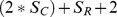, and the number of binary observations scales as 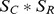. This means that there are relatively few observations per parameter for some of the less species-rich datasets we analyze, which leads to a potentially overparameterized model. Overparameterization might cause the BPNM to not be the best minimal model for explaining all aspects of ecological bipartite networks, and some parameters might be poorly estimated – necessitating caution when interpreting parameter values. The BPNM also has, to varying degrees, different numbers of parameters than the two other models we compare it to. We deal with these issues in several ways.

**Figure 2:**
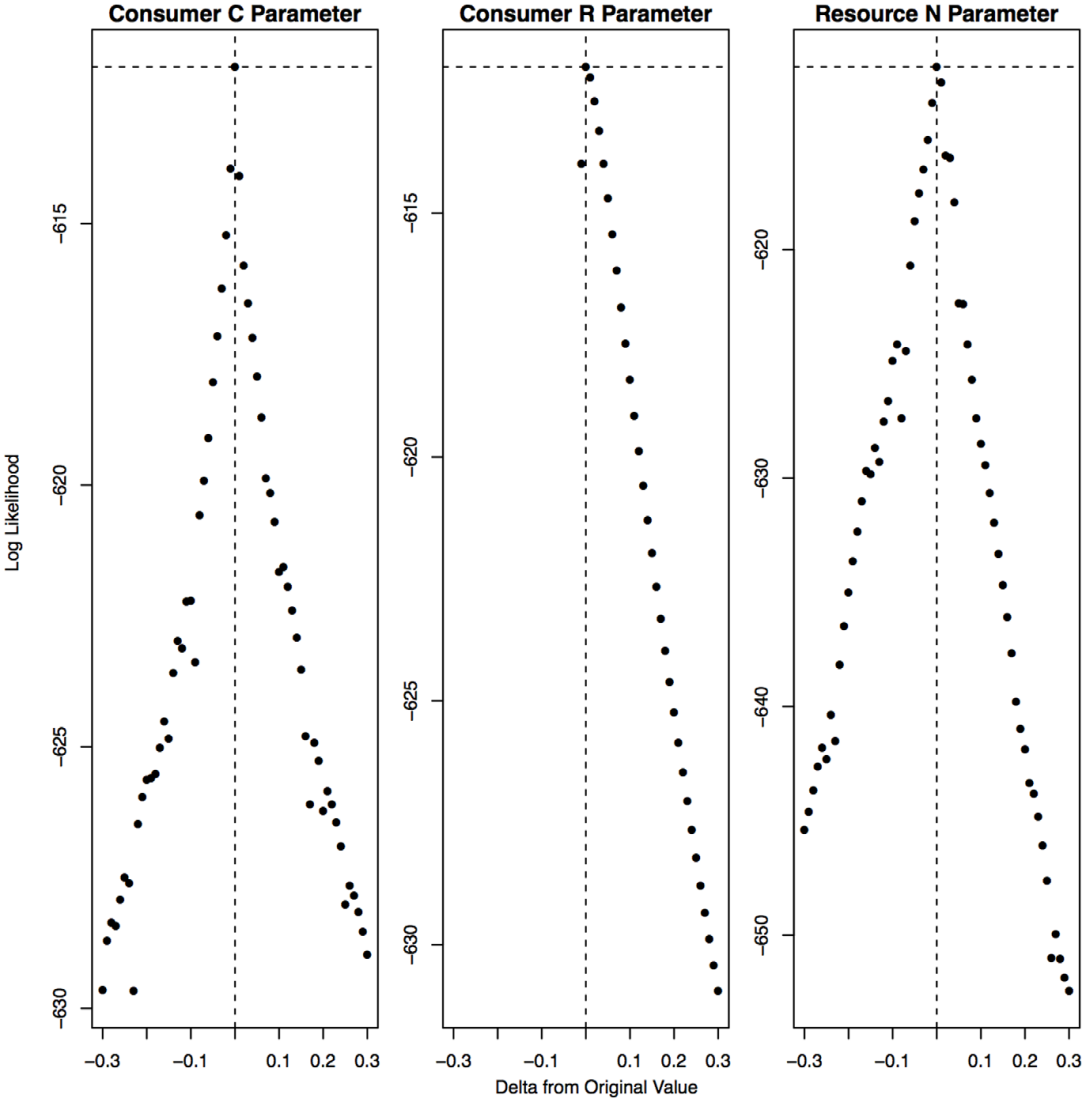
Parameter sensitivity for one randomly chosen consumer, and one randomly chosen resource species. The maximum likelihood value of the consumer parameters (c – left, r – middle) and resource parameter (n – right) is indicated by the vertical dashed line. Each parameter was perturbed across the continuum (x-axis) of −0.3 to 0.3 away from the maximum likelihood value (while holding all other parameters in the model constant). The corresponding likelihoods of each assessed parameter value are on the y-axis. Some parameters were already close to the value 0 and could not be changed to a smaller value, explaining why the distributions are not symmetrical in the centre plot.

First, since the different models in this analysis have different numbers of parameters, their relative performance on a single network is compared using AICc rather than by direct comparison of likelihoods. AIC_C_ allows the comparison of models with different numbers of parameters and includes a correction for small sample size. Second, we visually inspected the sensitivity of parameters by choosing several networks at random and, for each consumer and each resource, changing a particular parameter (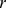,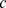, or 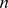) from −0.3 to +0.3 away from the maximum likelihood value, while holding all other parameters constant at their maximum likelihood values. One can assess sensitivity by analyzing the change in the likelihood surface moving towards and away from the maximum likelihood parameter. In Figure 2 we show examples of this for the 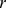 and 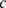 parameters of a randomly chosen consumer and the 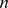 parameter of a randomly chosen resource species. In these three cases one sees the characteristic peak expected at the maximum likelihood value. Not all parameters are as sensitive as these, but even so, the problem of overparameterization is only likely to make our central conclusion below (that the BPNM outperforms other simpler models) overly conservative. Finally, previous work [26] explored an extension of the PNM, reducing parameters by making certain parameters functions of others, and when comparing models using AIC found that the PNM with its full parameter set consistently outperformed other, more minimal, simplifications of the same model.

### Other Models

We compared the performance of the BPNM against two simpler models. The simplest is a random model, in which every link occurs with constant probability 
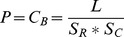
. A recent study suggested that a bipartite version of the cascade model [21] often provides a good model of mutualistic networks, and so we developed a probabilistic bipartite cascade model. Each consumer species is assigned a parameter 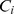 and each resource species is assigned a parameter 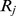. An interaction between consumer and resource is only possible if 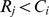 and the number of possible interactions 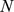 is the number of plant-animal pairs in which 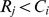. To force the expected number of links to be equal to the observed number of links we define 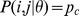 if 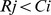 and 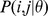 otherwise. The fixed link probability 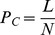 if 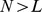 and 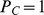 otherwise. As in the BPNM, we find the maximum likelihood set of model parameters 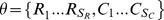.

### Niche Overlap Overview

The degree of dietary overlap between all consumers is a commonly measured structural property of ecological networks. In particular, ‘nestedness’ is a measure of the structure of a binary matrix, where, in this case, the rows represent resource species (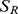), the columns represent consumer species (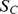), and a “1” in the matrix records the interaction between consumer 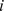 and resource 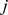. There are various metrics to calculate nestedness, all of which to some degree attempt to measure the niche overlap between consumers, or in other words, the extent to which the resource species of specialist consumers are proper subsets of the resource species of more generalist consumers (and vice-versa). To determine the BPNM’s predictive skill for the network aggregate metric of niche overlap, we first calculated the niche overlap of consumers in the empirical network using a standard nestedness metric, and compared that to two different approaches to calculate the niche overlap of the model-derived network.

### Empirical Niche Overlap

For our analysis we utilize the metric “NODF”, due to the transparency and statistical properties of the metric [30], [37]–[40] to measure the niche overlap of empirical networks. The original NODF algorithm calculates nestedness for the entire matrix, and independently for only rows (resources) or columns (consumers). The BPNM only calculates the niche range for consumers, and so we record only the consumer NODF value for each matrix (NODF_C_). Each bipartite network was sorted in descending order by both row and column marginal totals. The NODF value of a community of consumer species is a function of the paired overlap between all combinations of the 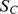
columns. The number of possible combinations is:



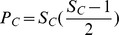
.

The paired overlap (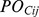) between any two (*i*,*j*) consumers is the fraction of resources taken by consumer 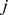 that are also taken by consumer 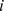. Note that the sorting described above means that it is always the case that the number of resources taken by consumer 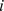 is always greater than or equal to the number of resources taken by consumer 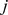. Defining 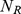 as the number of resource species taken either by consumer 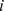 *or* 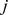, NODF then considers the paired overlap in consumer pairs, defining


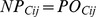
 if 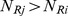, where 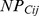 is not recorded (“NA”) in the event of 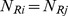.

We note that the original NODF algorithm records 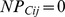 when two consumer’s feeding ranges perfectly overlap, yet it is debatable whether or not perfectly overlapping feeding ranges should result in a maximum deduction from nestedness, and thus for the purposes of this analysis we ignore those instances both in our estimating of empirical nestedness and calculation of overlap in model derived networks (although qualitative comparisons did not indicate a large discrepancy between the two).

NODF for consumers is then defined as the average of the 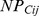 values: 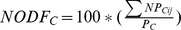; where 100 indicates perfect nestedness.

### Model Derived Niche Overlap

Because the BPNM provides a probability of every link, it is not possible to directly calculate standard nestedness scores for model-derived networks. To address this, we employed two different approaches. In the first, we simulated 100 random networks, where for any network the realization of any interaction between a consumer and resource was the result of a random binomial draw against the BPNM derived probability. We then calculated the niche overlap for each simulated network using the NODF algorithm as above, and compared the empirical niche overlap with the mean niche overlap across all 100 simulated networks.

We also note that like most structural properties NODF can be significantly sensitive to changes in only a few interactions across an entire network [37]. To avoid the problems with this due to simulating networks as above, we calculated a metric on the BPNM derived networks complementary to NODF. In the BPNM, the one-dimensional niche employed represents the resource dimension available to consumers. For this reason the NODF method of niche overlap measurement is complementary to measuring the overlap between the 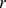 parameter of each consumer in a community. Thus, the consumer’s dietary range (“*r*” parameter) can be used to make a comparison between the niche overlap of the consumer community in the empirical network and the best-fit model derived community. To accomplish this we sort all 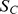 consumers by decreasing width of dietary range and for each consumer calculate the position of the 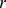 parameter cutoffs along the one dimensional niche axis:
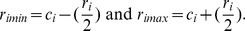



For any two consumers (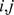), we calculate the percent of consumer *i*’s dietary range that overlaps with consumer *j*’s dietary range (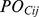, the continuous equivalent of the original NODF algorithm [40]), where 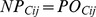 if 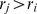, and where 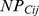 is not recorded (“NA”) in the event of 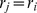.

The number of possible pairwise comparisons (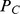) is the same as calculated above, and the overall niche overlap for consumers is then defined as the average of the 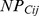 values:


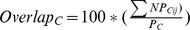
; where 100 indicates perfect overlap. Thus, 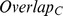 is a BPNM-specific method of measuring niche overlap on the same scale as 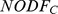.

### Connectance

We measured the size of the networks as 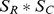, and the connectance of the empirical networks as 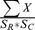. We measured the connectance of the model derived networks in two different ways. For the approach using 100 network simulations, we calculated model derived connectance as the average connectance of all 100 simulated networks. Avoiding simulating the networks resulted in the connectance metric 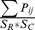.

## Results

### Replicating Consumer-resource Interactions

**Figure 3:**
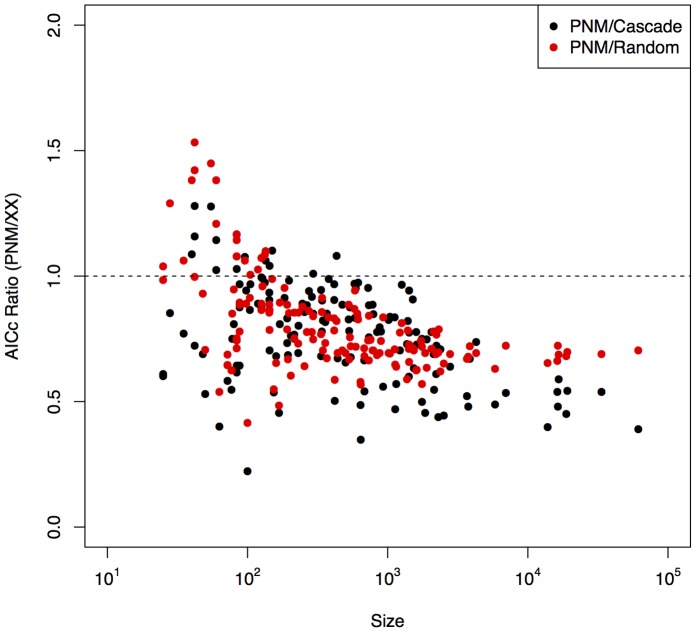
Performance of the random (red) and cascade (black) models as measured by AICc score. Results are presented relative to the PNM, where the x-axis shows the PNM AICc divided by the alternative model’s AICc. The random model outperforms the PNM only once, while the cascade model does so three times, all of which occur on the lower 50% of the size distribution of networks we analyze.

In Figure 3 we show that the probabilistic niche model consistently outperforms a random (red) model in all but 18 of our 151 networks. The BPNM outperforms a bipartite cascade model in all but 15 of the 151 networks (black points in Figure 3). The few cases where the BPNM model does not outperform the cascade model are those networks in the lower end of the range of sizes we analyze. Given the uniformly superior performance of the BPNM, the rest of the paper analyzes only the performance of that model in detail.

**Figure 4:**
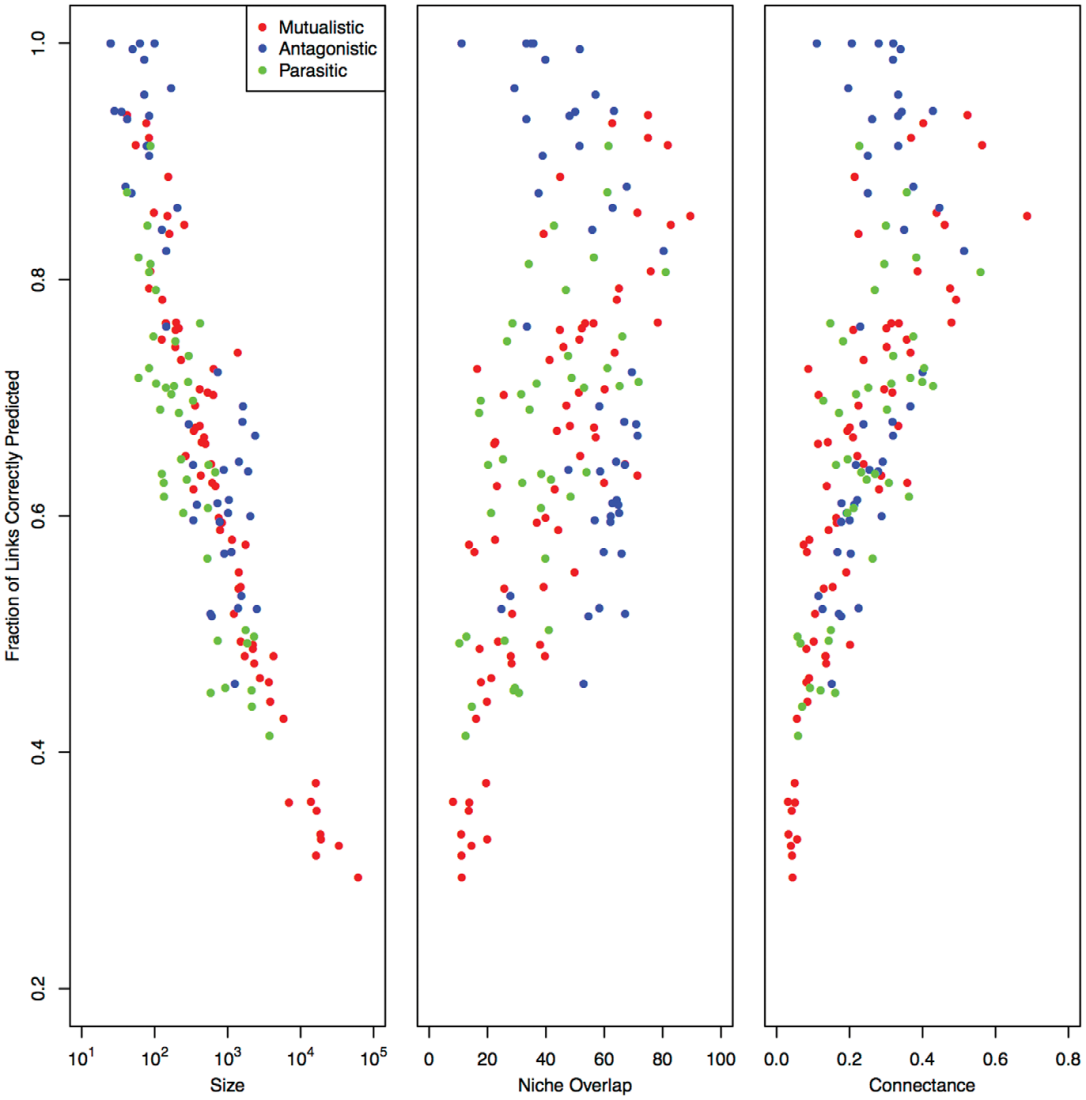
Left) Fraction of links predicted versus network size (#Resource Species * #Consumer Species); Centre ) Fraction of links (*f_L_*) correctly predicted (x-axis) versus empirical niche overlap (y-axis); Right) Fraction of links (*f_L_*) correctly predicted (x-axis) versus empirical connectance [#Links/(#Consumers * #Resources)] (y-axis). See Methods Summary for details on calculations of niche overlap.

In all networks, the expected number of links produced by the model, which is equal to the sum of the link probabilities, is very close to the number of interactions in the empirical dataset. As described and implemented previously by others [26], [31], we can thus use the fraction of links (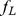) correctly predicted (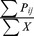) as a simple and easily understood measure of model performance comparable to previous work. We note, however, that if the total model predicted links is significantly different from the total number of links in the network then this measure of performance is insufficient, as a model predicting every link as realized would by necessity produce a perfect fit – while also incorrectly predicting every missing link. Figure 4 (left) shows how our measure of model performance (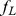) scales with network size. The decrease in 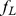 with size shows that a single niche dimension is sufficient for explaining individual interactions within small networks, but becomes insufficient for very large ones. This scale dependence in model fit exists for all three categories of interaction networks. Given the single dimension employed by the BPNM, the scale dependence in model fit is perhaps not surprising, as by random chance one expects larger networks to include greater heterogeneity in species trait distributions, and thus more dimensions for a niche space to be defined within.

### Replicating Consumer-resource Network Structure

A model that replicates the interactions between consumers and resources by necessity also replicates overall network structure, such as connectance, degree distribution, specialist-to-generalist consumer ordering, and niche overlap. One might assume that a model failing to replicate the interactions might also fail to replicate metrics of overall network structure, although analysis of empirical data has shown that temporal variability in individual interactions did not affect aggregated properties of network architecture such as nestedness [41]. We show that the BPNM is capable of reproducing three ecologically important aspects of network structure (connectance, niche overlap, and specialist-to-generalist consumer ordering) for all categories of networks regardless of how well the model does at predicting individual interactions within the network.

Niche overlap is a structural quality of a bipartite network and as a general ecological concept simply addresses the question: to what extent are the resources taken by specialist consumers proper subsets of the resources taken by more generalist consumers [37]–[40]? We focus on niche overlap (as measured by the nestedness metric NODF; see *Methods* for implementation details) for two reasons. First, it is claimed that consumers in mutualistic networks are more highly overlapping than antagonistic consumers due to differing coevolutionary pressures [3] and thus we might expect our model results to differ between these two interaction types. More importantly, we investigate niche overlap because it is closely related to the niche concept underlying the BPNM. The usual picture of a nested network is highly niche structured in a single niche dimension. In nested networks, a narrower niche (e.g. specialized consumer feeding range) is nested within broader ones (e.g. generalist consumer). In this view of nested networks, narrower niches have a high level of overlap with broader ones.

In keeping with the theoretical niche-structured assumptions of the BPNM, in Figure 4 (middle and right) we show that the BPNM performs best on the most highly nested and connected (across all networks Spearman’s correlation between connectance and nestedness = 0.80, p-value <0.001) empirical networks. This shows that connectance, the niche overlap, and the niche concept as implemented by the niche model are related, such that all highly nested (or connected) ecological networks are well-explained by a one dimensional niche structured model.

**Figure 5:**
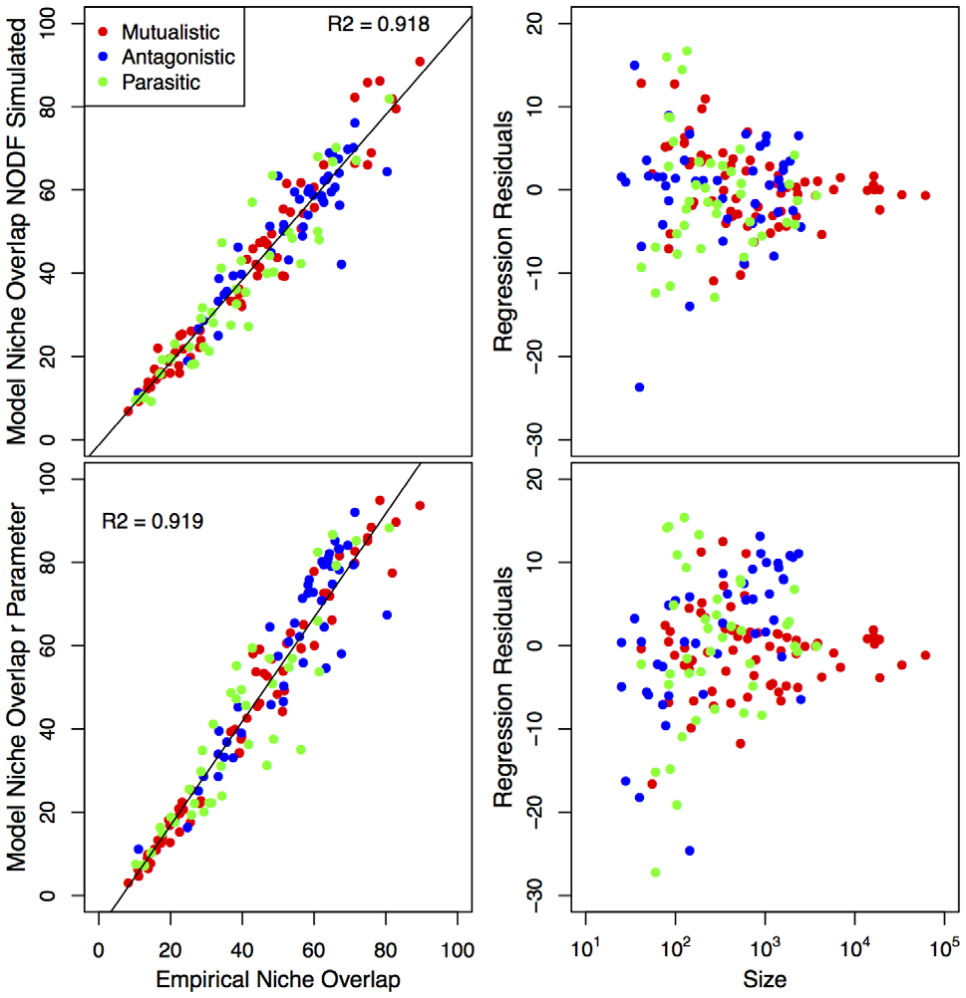
Top Left) Empirical niche overlap (as measured by NODF – see Methods) versus model derived niche overlap (as measured by the average NODF score of 100 simulated networks – see Methods); Top Right) Residuals about the opposing linear regression versus network size; Bottom Left) Empirical niche overlap (as measured by NODF – see Methods) versus model derived niche overlap (as measured by the overlap in consumer r parameters – see Methods); Bottom Right) Residuals about the opposing linear regression versus network size.

We further explore the relationship between niche structure and niche overlap in Figure 5 (top and bottom left) by plotting the niche overlap of the empirical network against the niche overlap of the model-derived network (see Methods Summary for our two alternative niche overlap calculations). Figure 5 shows the BPNM is capable of replicating the niche overlap structure even in networks that have a relatively low level of niche overlap and that, at the level of individual interactions, are poorly explained by the BPNM (have a low value of 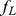), This means that regardless of how well the model performs at predicting specific links; it consistently replicates the niche overlap structure of the empirical networks. The linear regressions in Figure 5, and the relationship between the residuals about those regressions and the size of the network shows that model niche overlap most departs from empirical overlap in small networks with high degrees of overlap (Both regressions: *R* ^2^ = ∼0.92, *p*<2×10^−16^), and the range of the residuals decreases with network size.

**Figure 6:**
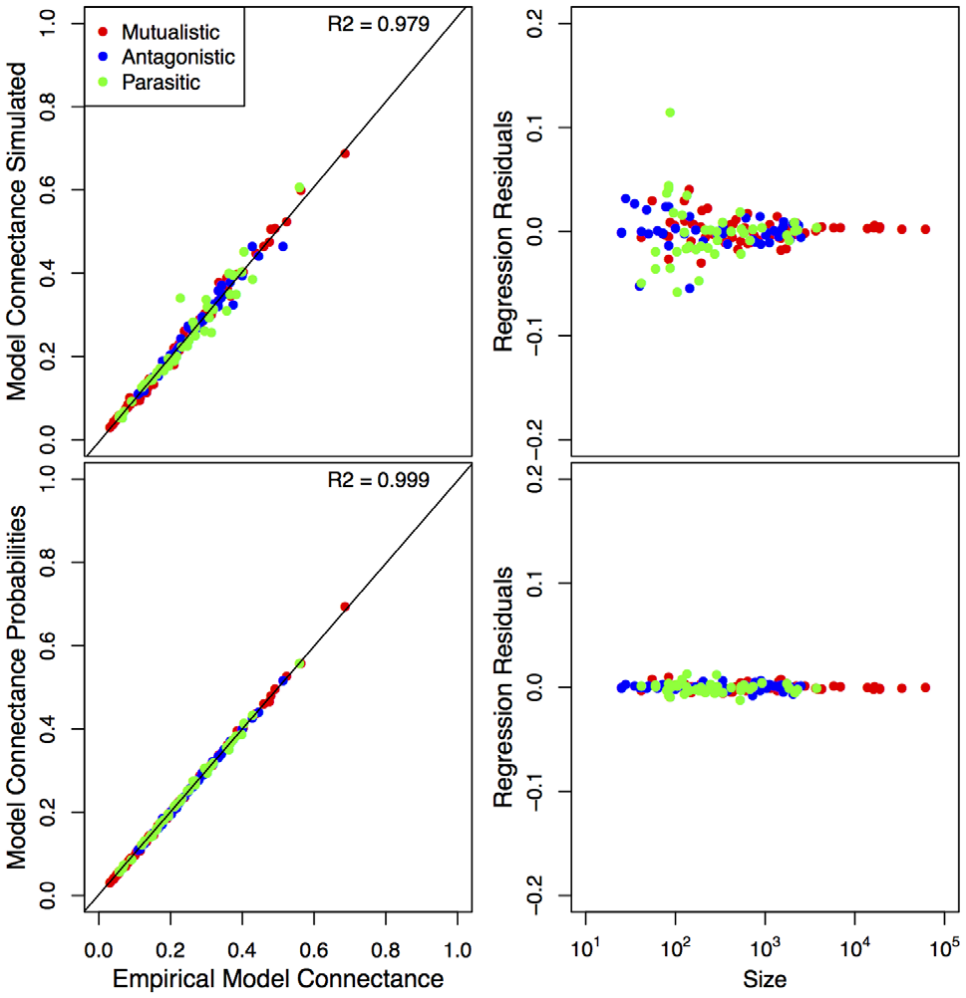
Top Left) Empirical connectance versus model derived connectance (as measured by the average connectance of 100 simulated networks – see Methods); Top Right) Residuals about the opposing linear regression versus network size; Bottom Left) Empirical connectance versus model derived connectance (sum of the model probabilities/network size – see Methods); Bottom Right) Residuals about the opposing linear regression versus network size.

Figure 6 replicates Figure 5, but shows the relationship between empirical connectance and two measures of model derived connectance (see Methods for details). In Figure 6 we show the same results to be true for connectance as for niche overlap, although even stronger. This replicates the strong correlation between those two measures in the empirical dataset. It can be argued that connectance is a more parsimonious descriptor of network structure than calculating more complicated niche overlap metrics such as nestedness. Yet, connectance and niche overlap need not be strongly related (except when approaching the limit of all interactions realized). We do not attempt to discern whether either is driving the results in Figures 4 and 5, as it is sufficiently interesting to note that the BPNM replicates both measures.

**Figure 7:**
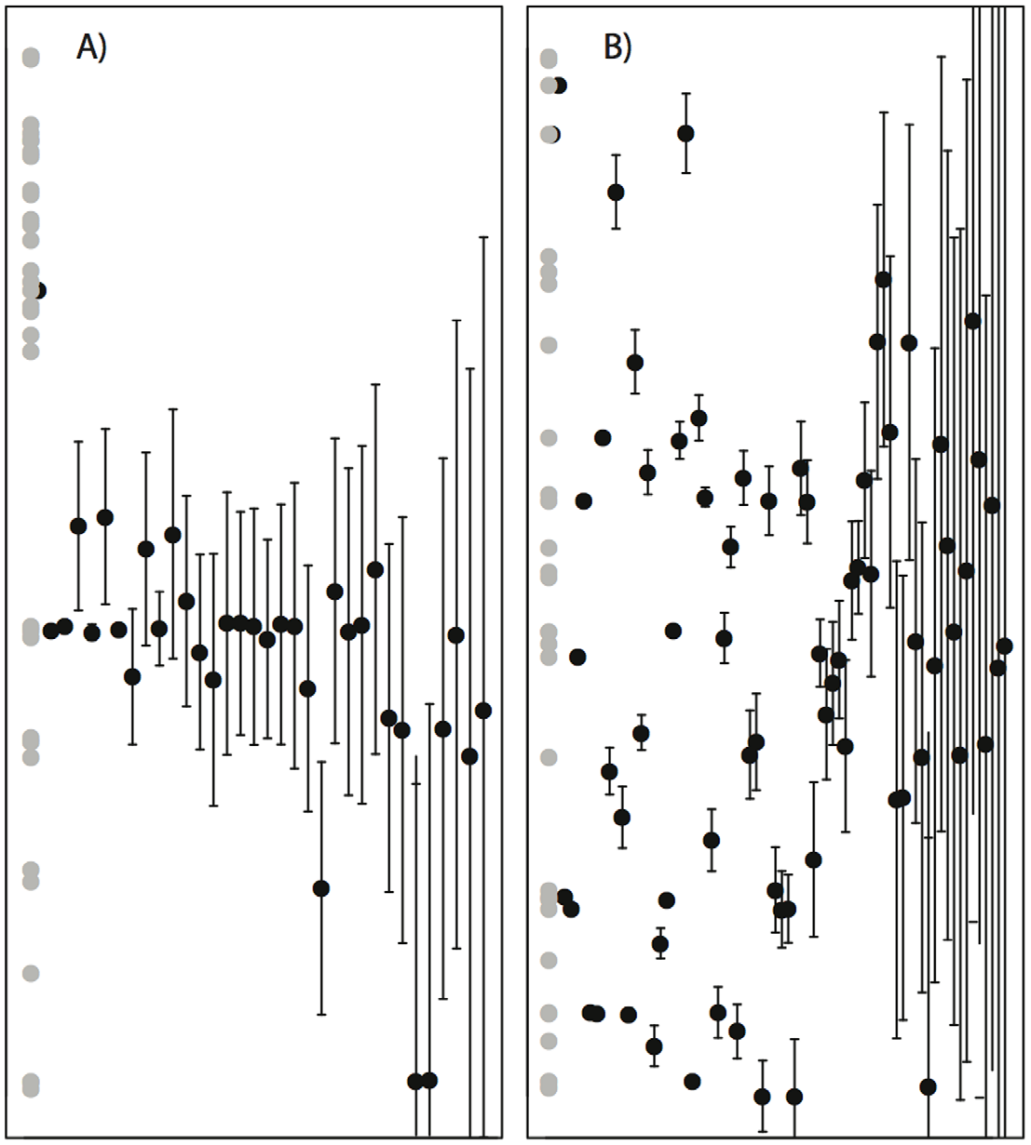
Plots of best-fit model parameters for two empirical networks. The y-axis represents the single dimensional axis assumed in the BPNM, scaling from 0 to 1. Light grey circles represent the niche positions (*n* parameters) of resource species along the single niche dimension. Black circles represent consumer niche center positions (*c* parameters), and the black bars represent the 97.5% range of consumer’s dietary widths (*r* parameters). Consumers have been ordered equidistant from one another along the x-axis, in order (left to right) from most specialized (consume the fewest resource species) to most generalist (consume the greatest number of resource species) according their rank witin the empirical interaction network. Left: An antagonistic network, where the best-fit model achieves 57% fraction of links (f*_L_*) correct. Right: A mutualistic network, where the best-fit model achieves 49% fraction of links (f*_L_*) correct.

Further, we show in Figure 7 that the BPNM, regardless of its ability to predict individual links, is capable of replicating another fundamental structural characteristic of ecological networks, which is the generalist to specialist consumer ordering. In Figure 7, the model-estimated niche axis position of the resources is plotted on the y-axis; while on the x-axis we have shown the model-estimated niche centroid (*c_i_*) and niche width (*r_i_*) of consumers in a mutualistic (Figure 7 right) and antagonistic (Figure 7 left) network. In both networks, however, we have ordered the consumers, from most specialized to most generalized, along the x-axis according to their *empirical* rank. It is immediately obvious that such an ordering closely matches the model ordering (measured as the consumer’s *r* value). Indeed, the average spearman’s rank-order correlation (0.90, 92% of p-values <0.05) across all empirical and model-derived networks shows this to be true.

### Differences between Mutualistic and Antagonistic Interaction Networks

**Figure 8:**
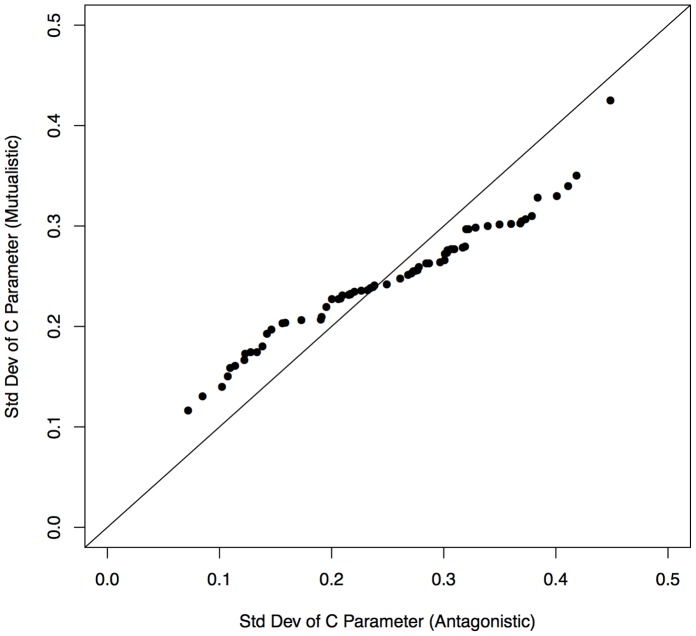
Q-Q plot of the standard deviation of consumer *c* parameter values in antagonistic (including parasitic) and mutualistic networks.

In Figure 7 (left) it is clear that the center of consumer’s niches in the antagonistic network is much more strongly clumped together than in the mutualistic network (Figure 7 right), although the distribution of the resources across the single axis remains fairly uniform. We examined whether this pattern recurred across all the networks by examining the distributions of niche positions (“*c*”) in all the networks and comparing the distributions between mutualistic and antagonistic (both predator-prey and parasite) networks. Distribution width was measured using the standard deviation of the *c* values, and the distribution of the standard deviation of *c* in mutualistic and antagonistic networks is significantly different (KS test, p = 0.029). Q-Q plots show the relationship between two probability distributions, where a one-to-one relationship on and x and y axes indicates the two distributions are the same. Thus, a Q-Q plot of the standard deviation of *c* for mutualistic and antagonistic networks (Figure 8) shows that *c* standard deviation of mutualistic networks is more likely to be intermediate-valued, corresponding to a near-uniform distribution. In contrast, the *c* standard deviation of an antagonistic network is more likely to be relatively small or large, corresponding to a more highly peaked distribution (as in Figure 7 left) or a distribution with multiple spread peaks respectively.

## Discussion

A recent study [38] suggested that a bipartite cascade model consistently gave as good or better results than a niche-structured model. Our results do not support this, instead showing that an optimally parameterized niche model almost always outperforms an optimally parameterized cascade model. This difference in results probably occurs because the rules used to assign niche positions and interaction niche parameters (center and width) previously used [38] are highly non-optimal. Importantly, when maximum likelihood parameter choices are made, the BPNM performs consistently better than the bipartite cascade model.

Santamaría and Rodríguez-Gironés [34] showed previously a combination of complementarity and cascade rules to be necessary to reproduce observed network properties. We show here that a unidimensional complementarity model is sufficient. One possible explanation for this discrepancy might be that the BPNM is probabilistic, while that of Santamaría and Rodríguez-Gironés is not. However, the discrepancy is more likely due to the fact that Santamaría and Rodríguez-Gironés used fixed uniform distributions for their model, while our results show that the niche value distributions within empirical networks are highly non-uniform.

We show that a simple one-dimensional probabilistic niche model is capable of closely replicating the structure of three broad categories of ecological bipartite networks, regardless of fundamentally different structures across these networks, and that the niche-structured model is a consistently better model than models without constrained niches. For the first time we show that two commonly identified themes within the ecological network literature, nestedness and niche, are closely related, and that using the niche concept is a simple and successful approach to modeling the structure of multiple types of bipartite ecological interaction networks.

Yet, we also find that there are clear differences in the niche structure of mutualistic and antagonistic networks. In particular, the distributions of niche centers are significantly different between mutualistic and antagonistic networks. It is likely that the consistent differences we show in the niche structure of mutualistic and antagonistic networks describe how mutualistic and antagonistic processes produce different constraints on species behaviors and interactions. This, in turn, directly influences our ability to predict and preserve earth’s natural systems. Interactions between species, and the structure of ecological networks those interactions create, both have important consequences for the functioning and robustness of ecosystems [2], [3]. The ability of a simple one-dimensional niche model to predict the structure of a wide range of ecological interaction networks demonstrates that common principles structure these systems. Discovering the order and consistent variability underlying what sometimes appear to be overwhelmingly complex systems is a vital step towards developing our ability to predict and preserve species, ecosystems, and their services.
